# Portable neuromodulation induces neuroplasticity to re-activate motor function recovery from brain injury: a high-density MEG case study

**DOI:** 10.1186/s12984-020-00772-5

**Published:** 2020-12-01

**Authors:** Ryan C. N. D’Arcy, Trevor Greene, Debbie Greene, Zack Frehlick, Shaun D. Fickling, Natasha Campbell, Tori Etheridge, Christopher Smith, Fabio Bollinger, Yuri Danilov, Ashley Livingstone, Pamela Tannouri, Pauline Martin, Bimal Lakhani

**Affiliations:** 1 Centre for Neurology Studies, HealthTech Connex, 13737 96th ave, Suite 204, Vancouver, BC V3V 0C6 Canada; 2 BrainNET, Health and Technology District, Vancouver, Canada; 3 Applied Sciences and Sciences, Simon Fraser University, Vancouver, Canada; 4 Centre for Brain Health (Radiology), University of British Columbia, Vancouver, Canada; 5 Department of Kinesiology, University of Wisconsin-Madison, Madison, USA; 6 Pavlov Institute of Physiology, Russian Academy of Science, Sankt Petersburg, Russia; 7 NeuroMotion Rehabilitation, Vancouver, Canada

**Keywords:** Traumatic brain injury (TBI), Motor function, Neuroplasticity, Portable neuromodulation stimulator (PoNS), Translingual neurostimulation (TLNS), Magnetoencephalography (MEG), Electroencephalography (EEG), Brain vital signs, Functional connectivity, Rehabilitation

## Abstract

**Background:**

In a recent high-profile case study, we used functional magnetic resonance imaging (fMRI) to monitor improvements in motor function related to neuroplasticity following rehabilitation for severe traumatic brain injury (TBI). The findings demonstrated that motor function improvements can occur years beyond current established limits. The current study extends the functional imaging investigation to characterize neuromodulation effects on neuroplasticity to further push the limits.

**Methods:**

Canadian Soldier Captain (retired) Trevor Greene (TG) survived a severe open-TBI when attacked with an axe during a 2006 combat tour in Afghanistan. TG has since continued intensive daily rehabilitation to recover motor function, experiencing an extended plateau using conventional physical therapy. To overcome this plateau, we paired translingual neurostimulation (TLNS) with the continuing rehabilitation program.

**Results:**

Combining TLNS with rehabilitation resulted in demonstrable clinical improvements along with corresponding changes in movement evoked electro-encephalography (EEG) activity. High-density magneto-encephalography (MEG) characterized cortical activation changes in corresponding beta frequency range (27 Hz). MEG activation changes corresponded with reduced interhemispheric inhibition in the post-central gyri regions together with increased right superior/middle frontal activation suggesting large scale network level changes.

**Conclusions:**

The findings provide valuable insight into the potential importance of non-invasive neuromodulation to enhance neuroplasticity mechanisms for recovery beyond the perceived limits of rehabilitation.

## Background

Acquired brain injuries, such as traumatic brain injury (TBI) and stroke, commonly result in significant long-term disability [1, 2], affecting critical abilities such as movement control. With increasing TBI survival rates from conflict zones, there is a growing push for novel therapies, which break down prior conventional limits of recovery [3]. Innovations in rehabilitation are beginning to integrate technology advances, particularly with the underlying concept of promoting enhanced recovery through neuroplasticity [4, 5]. In terms of clinical implementation, one approach to translation is to demonstrate individual-level technology advances first and then scale to larger clinical applications. This approach enables a research-driven framework in which practitioners can better address issues and challenges related to “false hope” by evaluating new treatments through research [6, 7]. This is particularly germane to the increasing broader societal awareness of neuroplasticity and the potential role of neuromodulation [8, 9].

Neuroplasticity generally refers to any adaptation process within the functional and structural aspects of the nervous system [10]. Neuroplasticity-related changes vary between healthy individuals compared to those with injuries or diseases and can be either adaptive or maladaptive in nature. Functional imaging, such as functional magnetic resonance imaging (fMRI), has been investigated extensively as a means to monitor and guide neuroplasticity-related recovery in rehabilitation [11]. Similar studies have expanded the multimodal imaging scope to include electro-encephalography (EEG) and magneto-encephalography (MEG) among others [12, 13]. Neuromodulation is increasingly being studied in terms of the ability to modulate neuroplasticity changes in the brain [4]. Neuromodulation through prolonged translingual stimulation has been shown to positively improved rehabilitation treatment outcomes, particularly balance and gait impairments, following brain injury [4, 14, 15].

In 2006, Captain (retired) Trevor Greene (TG) was attacked by a young male with an axe when on tour in Afghanistan and survived a severe open TBI. In 2016, D’Arcy et al. reported initial findings from an on-going investigation of TG’s unprecedented long-term motor recovery [16]. The overall objective continues to focus on pushing the limits of rehabilitation through neuroimaging and neurotechnology. TG’s injury largely involved motor function, with the axe impact damage extending along the axis of the mid-sagittal plane affecting frontal and parietal gray and white matter tissue, transecting the body of the corpus callosum (details below). TG’s rehabilitation objective involves recovering walking abilities, along with other movement-related impairments (e.g., rowing, as a former elite rower).

D’Arcy et al. [16] used longitudinal fMRI to monitor upper and lower limb motor activation recovery four times a year over three years (12 times total). Compared to a control, there was a statistically significant 5× increase in the extent of lower limb motor activation from the beginning of Year 1 to the end of Year 3. TG recovered clinically in parallel, as measured by movement abilities over the study duration, with the clinical scores correlating significantly to increased fMRI motor activation. Importantly, the findings were the first to utilize functional neuroimaging in order to demonstrate neuroplasticity-related recovery well beyond the conventionally adopted time limits (i.e., 6-months to 1 year). In TG’s case, rehabilitation progress had continued for more than six years post-injury, as measured by fMRI, at the time of the study.

### Current study overview

Since the 2016 study, TG has continued daily rehabilitation, but has experienced an extended plateau in the recovery of further abilities. In 2018, the current follow-up study began with the specific goal of investigating whether non-invasive neuromodulation, when paired with continuing rehabilitation, could help overcome the plateau and further push the limits of the recovery beyond 12+ years post-injury.

The study utilized translingual neurostimulation (TLNS) through the Portable Neuromodulation Stimulator (PoNS®; Helius Medical Technologies, Newtown, PA), a Health Canada Class II approved medical device that applies sequenced, non-invasive stimulation to the tongue. TLNS stimulation is generally believed to involve the trigeminal (CN-V) and facial (CN-VII) cranial nerves [17]. TLNS came to public attention in the book *The Brain That Changes Itself* [9]. The stimulation is hypothesized to converge on and modulate visual, vestibular, nociceptive, and visceral sensory signals through bottom-up cerebellar and brainstem pathways to produce neuromodulation effects [17–19]. There is initial evidence that stimulation of the trigeminal nerve activates networks involving sensorimotor and cognitive functions, with the possibility that the neuromodulation positively improves symptoms from various pathologies [20].

When paired with intensive physiotherapy (PT) in a multi-centre clinical trial, TLNS stimulation at both high- and low- frequency stimulation levels resulted in significant balance and gait improvements in mild-to-moderate TBI patients, with previous chronic refractory impairments [21, 22]. Subsequent examination of high- and low- frequency TLNS levels using high-density electroencephalography (EEG), healthy control, within-subjects, cross-over design, showed significant increases in alpha, theta, and attention-related spatial activity as well as a secondary intensity level exposure effect [23]. These recent results, in combination with several other prior related studies, have highlighted the need for functional neuroimaging to characterize the links between clinical effects and the underlying mechanisms of neuromodulation.

Given direct translingual stimulation of neurophysiological processes, magneto-encephalography (MEG) is an important neuroimaging modality because it enables spatio-temporal characterization of neural activation. In order to characterize single-subject PT-related improvements compared to translingual contributions to recovery, it is important to collect clinical and neuroimaging evidence over an extended PT plateau period (i.e., baseline) and then during the treatment onset of translingual neuromodulation (i.e., treatment). By comparing clinical and EEG measures of movement/motor improvements to MEG activation results, it is possible to evaluate the relative contribution of neuromodulation to neuroplasticity-related changes at the cortical level.

### Objectives and hypotheses

The objective of the current study was to characterize MEG changes related to motor function while TG underwent intensive PT alone for approximately one-year and then after TLNS was introduced for a 14-week trial period. MEG results were compared to both clinical and EEG measures of improvement.

Hypotheses: We hypothesized that PT + TLNS would lead to significant clinical improvements in movement abilities and that these would correspond to cortical network-level changes in MEG activation, as a function of neuroplasticity. MEG results were evaluated and confirmed using both contrast- and data-driven analyses to test for significant activation changes during the PT + TLNS period. A common trend in function was hypothesized to occur across all measures, showing no-significant change over the extended baseline of intensive PT alone, with an improvement during the intensive PT + TLNS period.

## Methods

### Nature of the injury

Detail on the nature of the injury is provided in the prior report [16]. In brief, TG was attacked on March 4, 2006 (at the time, 41 years old). He is right-handed, university-educated, and a soldier/journalist/writer. Research ethics approval was obtained from Simon Fraser University and the National Research Council. Captain Greene and his wife Debbie participate as full investigators in all aspects of the research (Note: They are both authors on this paper). The open severe TBI resulted from an attack with a crude axe. TG was leading a goodwill meeting with elders in the village of Shinkay, Kandahar, Afghanistan. As a sign of respect, the soldiers removed their helmets and laid down their weapons. A young male struck TG with the axe into the crown of his head with full strength, as a signal for a larger pending attack from the Taliban. Immediately after the engagement, TG’s vitals were stabilized through emergency care and he survived medivac extraction to Kandahar Air Field for advanced care. He transferred to the US Army Landstuhl Regional Medical Centre in Germany for neurosurgical treatment and induced into a medical coma. Once medically stable, TG was transported home to Vancouver General Hospital (Vancouver, Canada). Initial prognosis anticipated permanent vegetative state, but TG emerged from coma and recovered full consciousness after approximately 18 months. Following acute care, he was admitted to the Halvar Jonson Centre for Brain Injury Centre (Alberta, Canada) for a 14-month intensive rehabilitation program. Since then, TG has continued daily home-based rehabilitation with the main long-term objective of recovering ambulatory walking abilities and resumed an active writing career, which included publishing the book: “March Forth: An Inspiring True Story of a Canadian Soldier’s Journey of Love, Hope and Survival” [24].

The injury involved both penetration and rotational impact. The fracture to TG’s skull was approximately along the midsagittal plane, extending from the frontal bone posteriorly along the sagittal suture. There was both gray and white matter cortical tissue damage, extending laterally to the right frontal and left parietal lobes away from the midline and inferiorly to the lateral ventricle. The injury affected primary motor and premotor areas along with primary somatosensory and superior parietal areas. The injury depth affected the anterior cingulate gyri, corpus callosum (body and genu), and surrounding white matter tissue. See D’Arcy et al. [16], for a more detailed description along with MRI and fMRI imaging results. Visualization of the injury together with the summary analysis of the areas of greatest fMRI activation change over the three-year study is available in Additional file 1: Fig. S1.

### Experimental design

Similar to the prior study, we used a longitudinal design to evaluate motor activation changes over time. Clinical, EEG, and MEG data were acquired at regular intervals approximately every 3 months from July 2018 until August 2019. There were five time points in total, with three baseline (B1–3) and two treatment time points (T4–5). The treatment time points were collected halfway through the PT + TLNS program (7 weeks) and at the end of the program (14 weeks).

The baseline time points involved intensive PT alone and treatment time-points involved continued intensive PT + TLNS stimulation. Experimental parameters were kept constant across all time points. The timeline of clinical milestones relative to experimental assessments is indicated in Table 1.

**Table 1:** Timeline and milestones for clinical recovery, including date of initial injury and prior longitudinal studies with TG

Research timeline	Date	Clinical milestones
Phase 1: Motor fMRI Study (D'Arcy et al. 2016)	2006/03	Initial injury. *Prognosis: permanent vegetative state*
	2007/09	Fully conscious
	2007–2010	Intensive physical therapy
MRI 1	2010/05	Stands at wall-mounted bar without safety harness
MRI 2	2010/08	Takes steps inside parallel bar with harness and assistance
MRI 3	2010/11	No longer needed lift to get into MRI machine
MRI 4	2011/02	Stands and pivots with assistance
MRI 5	2011/05	Stands for 2 min with knee blocks
MRI 6	2011/08	Stands for 6 min with knee blocks and assistance
MRI 7	2011/11	Stands for 10 min with knee blocks assistance
MRI 8	2012/02	Stands for 30 s without knee blocks or assistance
MRI 9	2012/05	Sits without support
MRI 10	2012/08	Stands with walker
MRI 11	2012/11	Takes steps inside parallel bar with assistance
MRI 12	2013/02	Takes steps with walker with assistance
Phase 2: Current Study	2012–2013	PT with Lokomat device
	2014–2015	PT with ReWalk exoskeleton device
	2016–2018	Plateau in Recovery
	2018/04	Intensive physical therapy treatment begins
1st baseline (B1)	2018/07	2 min timed stand − moderate support FIST Score—13
2nd baseline (B2)	2018/10	1 min timed stand − moderate support FIST Score—19
3rd baseline (B3)	2019/04	FIST Score—12 Continued plateau in recovery coupled with lack of motivation and intensity
	2019/04	Physical therapy + translingual neurostimulation begins
1st treatment assessment (T1)	2019/05	20 min timed stand − moderate support FIST score—21
2nd treatment assessment (T2)	2019/07	20 min timed stand − minimal support FIST Score—33 Deep breathing improved—was able to blow up a balloon for the first time Renewed motivation and intensity to engage in therapy activities
	2019+	Ongoing physical therapy + translingual neurostimulation

### Neuromodulation

The PoNS® device consists of a light-weight portable controller worn around the neck and a stimulator with 143 gold-plated electrodes positioned to electrically stimulate the anterior dorsal tongue (1.5 mm diameter electrodes in a hexagonal pattern 2.2 mm apart; Kaczmarek, 2017). The patient holds the stimulator in place by applying upward pressure from the tongue. The stimulation level is adjustable and user-dependent, with increasing subjective intensity in discrete values from 1 to 60, which increase stimulation pulse length (μs) without any increase in electrical voltage levels (i.e., ensuring safe dosage control). PoNS® stimulation delivers pulses in triplets at 5 ms intervals (i.e., 200 Hz) every 20 ms (50 Hz), with a 17.5 V operating voltage and 440 μA current for each pulse. In accordance with a device level setting procedure, the user increases the stimulation levels to a comfortable sensation between the minimum perceptible level and the maximum tolerable level. The PoNS® procedure was developed from empirical psychophysical studies of optimal tactile sensation for comfortable long-term use.

### Study protocol

#### Clinical treatment and movement scores

The treatment program consisted of physiotherapy exercises for one-year (PT alone; B1–B3), followed by the same program paired with the PoNS® device for 14 weeks (PT + TLNS; T4–T5). Both the PT alone and PT + TLNS programs included six training days a week, with three training sessions a day. During in-clinic sessions, the therapist worked directly with TG. The PT alone program included three in-clinic days during each of the baseline testing visits and in-home training sessions between visits. The first week of the PT + TLNS training involved two out of three daily training sessions in-clinic to help familiarize TG with usage of the PoNS® device. The second week involved one out of three daily training session in-clinic. During weeks 3–14, TG took the device home and followed a physiotherapist-outlined program. Monthly check-in occurred to assess program goals and download usage data from the PoNS® device. Daily training sessions were divided into morning, afternoon and evening. Morning sessions consisted of a warm up (about 5 exercises working on upper body movements such as chin tucks, shoulder rolls, thoracic movements without the PoNS® device), balance (20 min of sitting balance work with the PoNS® device), gait (20 min sessions of standing exercises with the PoNS® device) and Breathing and Awareness training (BAT, 20 min of mindfulness/meditation with the PoNS® device). The afternoon session consisted of balance training, movement control (20 min of physiotherapy exercises without the PoNS® device) and gait. The evening session involved BAT.

Clinical movement scores included timed stand (up to 20 min) and the Function in Sitting Test (FIST) [25]. The timed stand was measured as the time TG stood independently in one place with assistance. The FIST is a 14 item, performance-based, clinical examination of sitting balance with demonstrated test–retest and intra- and interrater reliability [26]. The FIST bridges the gaps between simple observations about sitting balance/trunk control and balance measures more heavily weighted towards standing balance or gait ability. TG was asked to perform basic, everyday activities in a seated position with an examiner scoring his performance using a 0–4 point ordinal scale, with a maximum possible score of 56.

Upper limb motor control improvements were also a clinical priority. As described in the prior report [16], upper arm function has recovered to a higher functional level than lower limb abilities. For example, TG performs many common daily upper limb tasks independently. However, on-going rehabilitation has also focused on improved upper limb function as spasticity has continued to limit functional recovery. With respect to spasticity, motor control, and movement abilities, TG has a clear right > left functional asymmetry. While previously right hand dominant, he performs most functional tasks with his left hand. Given the technical constraints of MEG and the established pattern of upper limb motor control impairment, basic left and right responses were selected to investigate corresponding changes in both EEG and MEG activation over time.

#### EEG—motor function

Using a modified pre-established protocol [27], motor EEG data were also recorded at five time points: B1, B2, B3, T4, T5. Data were collected using a 32-channel recording system (g.Nautilus g.LADYbird, g.tec medical engineering, Graz, Austria) at a sampling frequency of 500 Hz. The design of the motor task mirrored MEG data collections, with TG responding using a custom designed button pad at a self-guided pace (approximately every 2–4 s) with all four fingers (in sequence, digits 1–4). At each visit, TG performed three × 2.5-min motor sessions with each hand.

For each EEG recording session, data were manually cleaned to reduce artifacts and to ensure task compliance. Data segments containing major artifacts (muscle activity and large movement artifacts in particular) were discarded. Button press events that occurred out of sequence (for example, multiple buttons pressed simultaneously) were also discarded. After identifying clean EEG segments, an average of approximately 70 click events per hand (~ 66% of available events) remained for each recording session.

Following artifact rejection, data were processed to extract event-related desynchronization/synchronization (ERD/ERS) activity [28] for the motor task. To identify the frequencies of interest for TG, time–frequency analysis (time locked to the click event and averaged across trials) was performed from 5 to 35 Hz for each recording session. For TF analysis, a Laplacian spatial filter was applied to the electrodes in the contra-lateral motor area. (Laplacian for left hemisphere centered at C3 with [FC5 FC1 CP5 CP1] as the surrounding electrodes; mirrored electrodes for the right hemisphere.) TF analysis identified the high beta range as demonstrating robust ERD/ERS activity. Subsequently, all electrodes were filtered from 24–30 Hz, corresponding to the most active frequencies in TF analysis.

Data were further processed with an optimal spatial filter technique [29] which uses gradient descent to find the linear combination of channels that maximizes the power ratio between two conditions (in this case, the post-movement period where high power (ERS) is expected and the pre-movement period where low power (ERD) is expected). This technique, while not traditional, was chosen to maximize ERD/ERS signal quality by providing some targeted source localization. For each recording session, EEG electrodes in the contra-lateral hemisphere were used to calculate the channel weights that maximized beta power in the post-movement period (from 0 to 0.5 s after button press) relative to the pre-movement period (from -1 to 0 s prior to button press). (For the left hemisphere, input channels were [AF3 F7 F3 FC5 FC1 T7 C3 CP5 CP1 P7 P3]; mirrored electrodes for the right hemisphere. The Laplacian described above was used as the ‘initial guess’).

To quantify ERD/ERS activity for comparison between sessions, beta rebound (magnitude of beta ERS following the movement event) was selected as the relevant output measure. Beta rebound is typically measured relative to resting activity, but due to the sequential nature of the finger movement task, there was no baseline rest period for each event. Instead, the period immediately prior to button press (during movement, while ERD is occuring) was used as the baseline. Beta rebound was calculated as (max ERS − min ERD)/(min ERD).

To determine the reliability of the beta rebound output measures for each session, 5000 bootstrap iterations were calculated using random sampling of epochs with replacement. To test the significance of changes during treatment, the difference between each treatment session and the overall baseline was compared using a weighted contrast: T − 1/3 (B1 + B2 + B3). Bootstrap outputs were resampled (100,000 iterations) to determine the overall distribution for the contrast hypothesis, then the significance of the result was determined using a percentile test.

#### MEG

MEG data were recorded at 5 time points: B1, B2, B3, T4, T5. Data were collected at a sampling frequency of 1200 Hz in a magnetically shielded room using a 275-channel MEG system (CTF systems; Coquitlam, Canada), and the head position was continuously tracked. The data recording was performed while TG was in a seated position during four 5-min motor task data acquisition (2 per hand in a randomly assigned order). In the motor task, TG was instructed to press a button (Lumitouch, Photon Control Inc., Burnaby, Canada) at a self-guided pace, approximately every 2 to 4 s in sequence with digits 1 to 4 alternating between both high right and left hands (Note: M/EEG resting state, with eyes open- and closed-10 min sessions, were also recorded and analyzed in a separate study).

Prior to the MEG data collection, the head shape was digitized using a Polhemus FASTRAK digitizer for co-registration of MEG data with his anatomical MRI. The anatomical MRI was segmented using Freesurfer. The gray/white matter boundary mesh was down-sampled to 4 K vertices and brain activity was estimated for each vertex.

For each recording, noise segments with muscular artifacts or head motion exceeding 5 mm from the median head position during the recording were rejected. Then, independent component analysis (ICA) was computed and artifactual components were discarded. Trials free from noise segments spanning from − 0.5 to 1.5 s relative to button press onset were grouped for each session and condition.

To estimate brain activity during the motor task, an event-related Dynamical Imaging of Coherent Sources (DICS) beamformer [30] was calculated using FieldTrip [31] to localize the motor signal. Similar to the EEG analysis, the post-movement ERS ‘rebound’ period (from 0.25 to 1 s after button press) was contrasted against the pre-movement ERD period (from 0.75 to 0 s prior to button press). First, the complex Fourier spectra were calculated in the same high beta range (centralized at 27 Hz with a 5 Hz taper parameter) where motor activity was most prominent in the EEG analysis. Next, the inverse filter was computed using data from both the ‘rebound’ and ‘active’ periods, then applied to the two conditions separately. Finally, the contrasted motor activity map was calculated for each trial according to the formula (post-movement ERS − pre-movement ERD)/(pre-movement ERD).

To test the significance of differences between sessions, Partial Least Squares (PLS) analysis was used [32, 33]. PLS is a multivariate statistical approach based on singular value decomposition. In this study, we used both Non-Rotated Task PLS (referred to here as ‘contrast-driven’ PLS) and Mean Centering Task PLS (referred to here as ‘data-driven’ PLS). Multiple analyses were used to confirm common MEG activation changes for right hand (RH) and left hand (LH).

The contrast-driven method enables testing of specific hypotheses about the contrast between conditions by setting a hypothesis driven design matrix. A specific contrast pattern is provided as an input and PLS identifies the singular value and salience of the input contrast. To test significance, a series of permutations was run by permuting trials across sessions. A single p-value is rendered to mitigate against multiple comparisons. In our case, to identify whether contrast-driven changes were robust against variation in the contrast pattern, the contrast-driven analysis was run three separate times using timed stand, FIST, and EEG beta rebound (see above) as the input contrasts.

The goal of the data-driven method is to automatically decompose the multidimensional data into ‘latent variables,’ which describe the maximum variance between sessions. Each latent variable is composed of three elements: a contrast pattern (describing change between sessions); a singular value (describing the strength of the change); and a vector of saliences (describing the brain vertices expressing the change). As with data-driven PLS, permutation was used to test the significance. A single p-value per latent variable is rendered to mitigate against multiple comparisons.

PLS also includes bootstrapping, which enables visualization of robustness of change across brain vertices. The output can be interpreted as z-scores that demonstrate the spatial distribution of differences between sessions. For all contrast- and data-driven cases, bootstrapping was used to generate spatial visualizations of the activity changes.

## Results

### Clinical treatment and movement scores

**Fig. 1:**
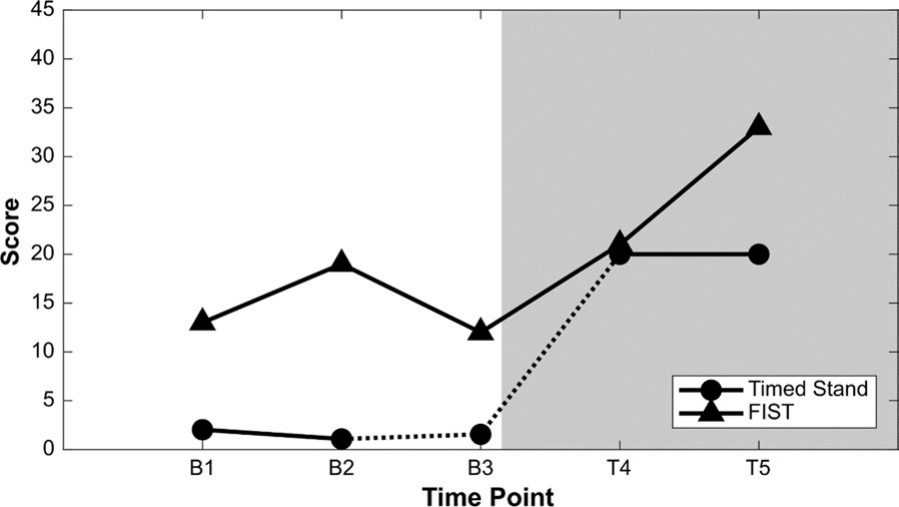
Changes in clinical outcome measures for Timed Stand and FIST tests. The shaded area denotes the TLNS treatment period. Note: Timed Stand test was not completed at B3 time point, dotted line indicates B3 measure as the average of B1 and B2. Shaded area represents onset of treatment.

Figure 1 shows changes in clinical outcome measures for both timed stand and the FIST. TG’s initial timed stand was < 30 s without support. Six months prior to B1, TG was unable to stand supported for more than 5 min. During B1–3, TG was unable to stand independently for more than 2 min. Timed stand performance increased quickly over T4–5. After 7 weeks with PT + TLNS, he stood for 20 min with one aid (therapist) providing moderate support. At 14 weeks, he stood for 20 min with one aid providing minimal assistance.

In addition to timed stand increases, the change in difficulty of position reflected improvements in motor control. For example, TG progressed from moderate support (two hands on the therapist’s shoulders) to minimal assistance. Free arms in the standing position requires increased motor control for the core and trunk: initially, TG relied on his arms to support his upper body. Over time, he was able to activate his extensors and core to maintain stability while moving his limbs and body, outlining improvements in motor function and control. This increased trunk control was similarly demonstrated with the FIST results.

Baseline FIST scores were recorded at B1–B3 (July 2018, October 2018 and April 2019) and scored as 13, 19 and 12. Treatment FIST scores T4 (7 treatment weeks) T5 (14 treatment weeks) were 21 and 33, respectively. A change in score ≥ 6.5 points on the FIST is considered clinically meaningful [34]. In addition to the clinical measures above, a range of qualitative improvements were observed and reported by TG and his wife, including a noteworthy reduction in TG’s symptoms related post-traumatic stress disorder (PTSD).

### EEG—motor function

**Fig. 2:**
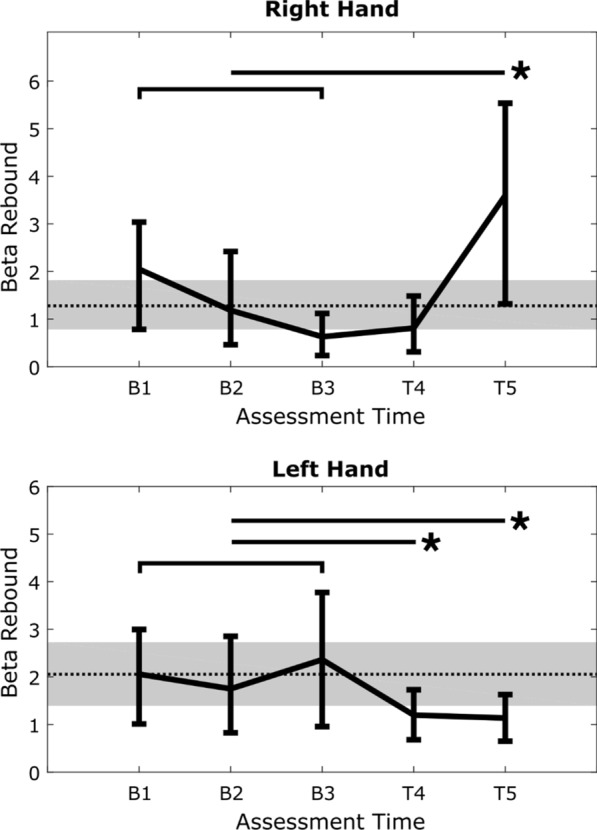
EEG motor function changes (contra-lateral beta rebound, mean and 95% confidence interval) for both right and left hand movement over three baseline and two treatment periods. Shaded area denotes the 95% confidence interval for the mean of the three baseline measurements. *p < 0.05

Figure 2 illustrates the motor-related contra-lateral beta rebound activity changes for right and left hand movement over baseline and treatment periods. Right hand activity changes indicate an increase in contra-lateral beta rebound (p = 0.0489) for T5 relative to baseline. Left hand activity changes indicate a decrease in contra-lateral beta rebound relative to baseline for both T4 (p = 0.0480) and T5 (p = 0.0289).

### MEG

**Fig. 3:**
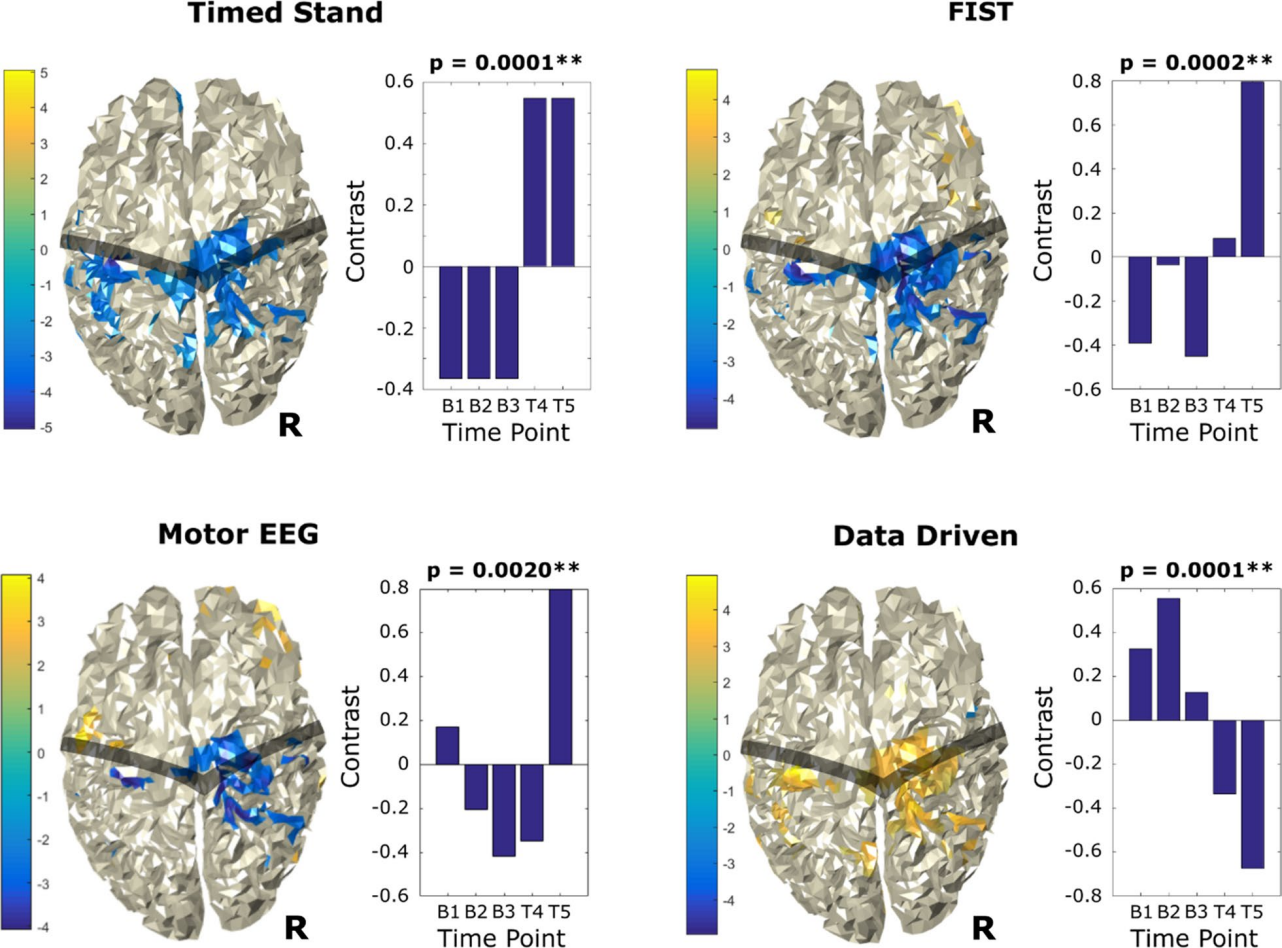
MEG activation changes and bootstrapped Z-score plots for right hand movement over baseline (B1, B2, B3) and treatment (T4, T5) time points, revealing convergent activation results across all contrasts and data driven analyses. Top left: Timed Stand contrast-driven PLS. Top right: FIST contrast-driven PLS. Bottom left: Motor EEG contrast-driven PLS. Bottom right: data-driven PLS. Central Sulcus region is shaded for reference. *p < 0.05, **p < 0.01

**Fig. 4:**
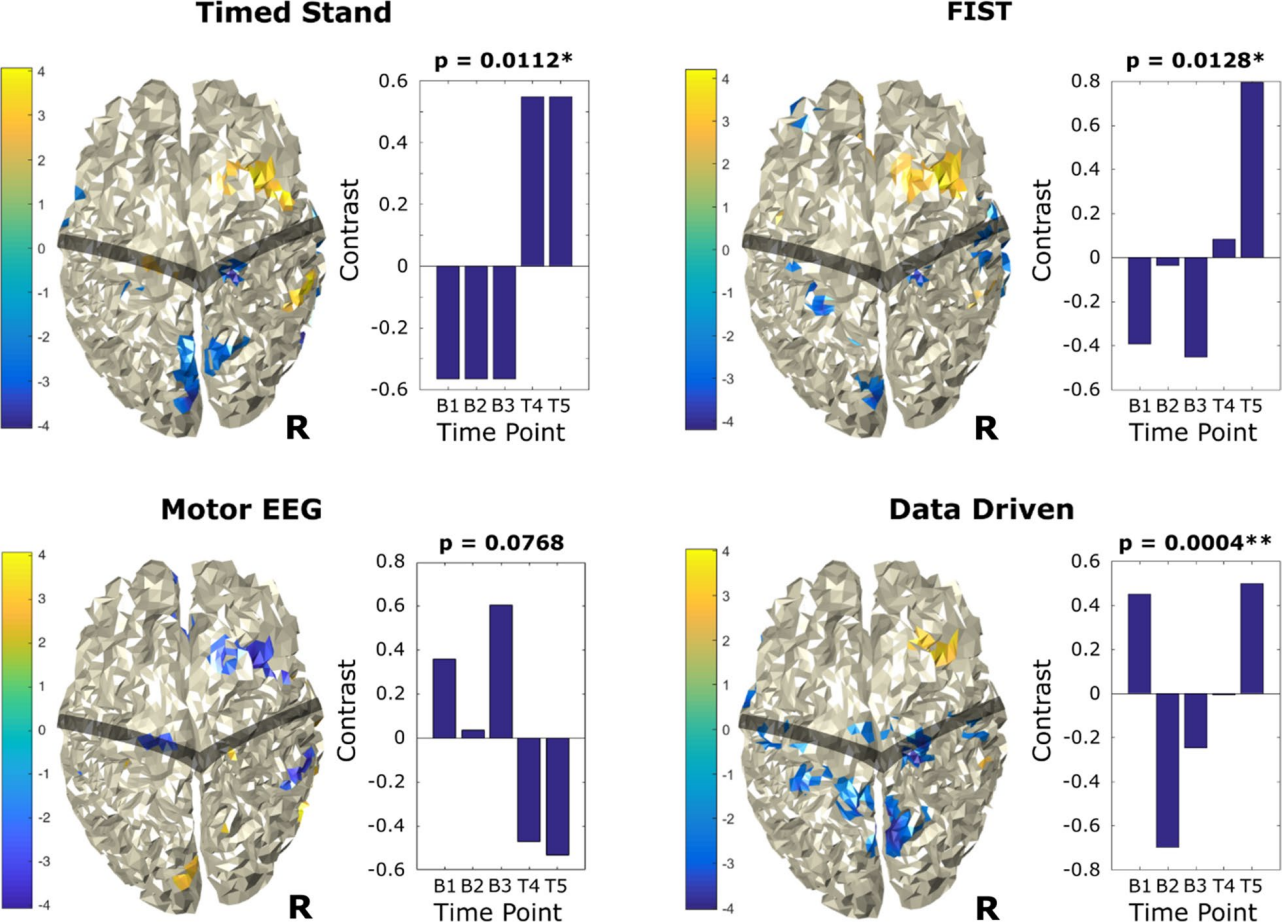
MEG activation changes and bootstrapped Z-score plots for left hand movement over baseline (B1, B2, B3) and treatment (T4, T5) time points, revealing convergent activation results across all contrasts and data driven analyses. Top left: Timed Stand contrast-driven PLS. Top right: FIST contrast-driven PLS. Bottom left: Motor EEG contrast-driven PLS. Bottom right: data-driven PLS. Central Sulcus region is shaded for reference. *p < 0.05, **p < 0.01

Figures 3 and 4 show significant MEG activation changes for right- and left-hand movement over baseline and treatment, revealing convergent activation results across all contrast-driven and data-driven analyses. Displayed spatial maps are based on the z-score output of PLS bootstrapping. A z-score threshold of ± 2 has been applied to specifically highlight the brain regions where change across sessions was most robust. Note that, while the data-driven analyses identified multiple significant contrasts, for simplicity only the most significant contrast for each hand is shown. The position of the central sulcus is highlighted as an anatomical reference point.

For right hand movement (Fig. 3), all contrast-driven analyses indicate a general decrease in beta activation of the motor region of the right (ipsi-lateral) hemisphere (for treatment sessions relative to baseline). The data-driven contrast also highlights this same activity profile (although, as expected, the contrast and colour grading are inverted). For left hand movement (Fig. 4), all analyses indicate an increase in beta activation of the pre-motor area of the right (contra-lateral) hemisphere (for treatment sessions relative to baseline). Some analyses also highlight a small area of decrease in the right (contra-lateral) motor region. It is particularly important that multiple analyses identified the same brain regions, indicating that the activation changes in these regions were robust.

## Discussion

The study findings supported the primary hypothesis that PT + TLNS treatment would lead to observable movement ability improvements, despite having reached an extended clinical plateau 12 + years following TGs severe TBI (Table 1). EEG motor activation revealed significant differences in both the right and left hand, with an increase in right-hand beta rebound and decrease in left-hand beta rebound. Importantly, the EEG results pattern was accompanied by corresponding MEG activation changes (Fig. 1). Right hand MEG activation changes showed a significant decrease in interhemispheric bilateral post-central regions, with the largest extent in the right hemisphere (Fig. 2). Left hand MEG activation changes resulted in a significant increase in right frontal superior/middle regions (Fig. 3). It is noteworthy that consistent MEG results were present across contrast- and data- driven analyses, indicating robust network-level effects occurred across the analyses.

Role of neuromodulation: The PT + TLNS combination resulted in demonstrable clinical improvements overcoming the prior 1-year rehabilitation plateau. It is important to highlight that TG was 12 + years post-injury and continued intensive daily rehabilitation efforts despite experiencing an extended plateau. In this instance, prior rehabilitation efforts included standing interventions such as parallel bars, overhead body-weight supported robotic gait assistance (Lokomat®), and the use of a robotic exoskeleton (ReWalk®), with none resulting in an improved timed standing test. As described anecdotally by one of the co-authors, Debbie Greene: “If the key factor was rehabilitation, rather than the PoNS, Trevor’s years of intensive rehabilitation would have shown it.” The reported clinical gains, while seemingly small, are therefore substantial, particularly when considering the relatively short 12-week period of PT + TLNS in comparison to the recovery plateau following 12 + years of ongoing PT after injury. In fact, TG continues to make progress after this case study which will be documented in future work.

EEG was used to evaluate upper limb motor function and compare right- and left- hand activation changes between baseline and treatment periods. Prior work has shown that EEG can be used as an objective score for upper limb motor function in healthy individuals along with stroke survivors, with a high predictive accuracy for clinical motor assessment scores [27]. In TG’s case, for right hand activity there was an increase in contra-lateral beta rebound during the treatment period (compared to baseline) and a decrease in the same measure for left hand activity. TG has a right > left hand movement impairment, with higher spasticity in his right hand. Consequently, the right > left movement asymmetry is particularly interesting given the MEG activation results, as it appears to suggest the potential involvement of interhemispheric inhibition [35].

In the prior study, we noted that the relationship between fMRI activation changes and neuroplasticity-related improvements remained to be fully characterized. Although still far from understood, the current MEG (and EEG) results provide important additional insights. MEG has been shown to be sensitive to neurophysiological biomarkers of upper limb motor function following acquired brain injury [36]. Beta MEG activity has been identified as a key marker of motor control [37]. Examination of Fig. 2 shows a significant reduction in interhemispheric beta (27 Hz) rebound activation in right hand movement, with the greatest reduction in the ipsi-lateral post-central gyrus. One interpretation for the reduced ipsi-lateral activation relates to a reduction in interhemispheric inhibition during increased right-hand movement abilities. While the injury transected the anterior body of the corpus callosum, the results suggest connectivity corresponding with prior fMRI activation in regions immediately posterior to damaged structures (Additional file 1: Fig. S1). The left hand had greater functionality than the right. In this case, increased beta rebound activation was detected in the contra-lateral frontal regions, suggesting further improved motor function.

However, this interpretation of neuroplasticity-related network level change remains to be confirmed. Two future steps will be necessary (at a minimum) to further demonstrate such network-level changes: (1) Confirm the task-based results using connectivity analyses of resting state data (both fMRI and MEG), which will be reported in future studies; and (2) the integration of transcranial magnetic stimulation (TMS) to evaluate and confirm the relative role of functional regions and interhemispheric interactions that have been identified.

The neural mechanisms that underlie PoNS®-related neuroplasticity effects are still being characterized. Frehlick et al. [23] used high-density EEG to investigate PoNS® effects and showed significant increases in alpha and theta signal power following a single session. Danilov et al. [17] provided an in-depth review of the hypothesis for translingual stimulation inducing neuroplasticity. Through non-invasive stimulation of the trigeminal (CN-V) and facial (CN-VII) nerves, the current concept predicts excitation that leads to functional changes across a range of brain systems. In support of this, neural activity changes have been detected in fMRI [19, 38]. It is postulated that intensive activation of the brainstem and cerebellar structures initiates a cascade of activation through direct projections and collateral connections, and/or through modulated biochemical signalling mechanisms. This phenomenon appears similar to well-known processes such as long-term potentiation (LTP) and depression. Importantly, when paired with PT (and possibly other forms of intervention), synaptic plasticity may arise through activity-dependent processes to integrate adaptive changes at the molecular, cellular, regional, and systemic levels, which in turn result in a host of improvements in sensory-motor functions, cognitive functions, and behavior [17].

With respect to the hypothesized mechanisms above, it is noteworthy that TG’s injury did not involve brainstem and cerebellar structure, but rather cortical gray and white matter tissue. In spite of the extensive nature of the lesion, TLNS induced significant neuroplasticity-related improvements. While not the focus of the current paper, resting state fMRI/MEG data were also collected in order to analyze functional connectivity changes at a network level in future reports.

Several caveats must be highlighted from the current study. First, like-for-like comparison of EEG and MEG results is limited by differences in recording technologies and analysis methods. The MEG analysis was based on DICS beamformer source localization. With only 32 channels, EEG source localization was not conducted. To achieve some targeted source localization of beta ERD/ERS activity in EEG, optimal spatial filtering was applied, which does not lend well to topographical visualisation. Second, EEG and MEG analyses focused on beta motor activity (which is often studied alongside alpha motor activity). Beta ERD/ERS was found to be most robust in the initial EEG time–frequency analysis, which may be a limitation of the sequential motor task. With limited ‘baseline’ rest between movement events, it was most appropriate to measure post-movement ERS relative to pre-movement ERD. Alpha activity, classically described as having less robust ERS [28], may not be well suited to this analysis. This case study demonstrates that TG’s clinical functional gains are related to improvements in both neural structure and function, however it is difficult to isolate neural recovery or neural compensation as the true underlying cause.

In addition, the current results are based on a longitudinal case study and required further support from studies with larger sample sizes. Furthermore, the results report on two time points during PoNS® treatment and further time points will enable examination of whether the improvements are sustained over the return to rehabilitation within the home setting. Finally, the generalizability of the results may be limited given the unique nature of TG’s case. It will be important to examine individual variance and percentage of outcome success to better understand key factors that enable neuromodulation of neuroplasticity to be utilized more widely in rehabilitation.

## Conclusion

With the increasing role of neuroplasticity in pushing conventional rehabilitation limits, imaging and technology advances are playing an important role in assessment and treatment, respectively. The current study reduces this concept down to a concrete demonstration following 12+ years, where TG’s example represents a proof-of-concept, the finding from which can be scaled to wider clinical applications for patients with chronic rehabilitation plateaus. TG’s extended rehabilitation plateau was disrupted through combined PT and neuromodulation, as measured clinically, with EEG motor function, and MEG cortical activation changes.

## Supplementary information

**Additional file 1: Figure S1.** Part A Five regions—randomly labelled A-E—of greatest fMRI activation change over the three-year study duration from Phase 1, showing increased activation in areas immediately posterior to damaged structures. Co-registered functional data were concatenated into a 4D volume and passed to FSL MELODIC for independent components analysis along the 4th dimension, using automatic dimensionality estimation. Resulting temporal components were averaged into years 1–3 and compared using a paired t-test. Spatial distribution maps localized the regions of greatest changing brain activity. FSL Cluster was used to extract “neuroplasticity clusters” from the lower limb IC3 map. (Refer to Part B for activation changes over time for each of these regions). All activation is thresholded for p<0.05 (corrected). Part B: Activation changes over the three-year study duration for the five clustered regions (labelled A–E) of largest overall fMRI change (shown in Additional figure Part A). Blue lines represent the activation for each voxel within the cluster, red lines show the average activation of all voxels, and the line of best fit indicated in black to show the general trend over time.

## Data Availability

The datasets used and/or analysed during the current study are available from the corresponding author on reasonable request.

## Abbreviations

DICS: Dynamical imaging of coherent sources
EEG: Electro-encephalography
ICA: Independent component analysis
FIST: Function in sitting test
fMRI: Functional magnetic resonance imaging
LH: Left hand
LTP: Long-term potentiation
MEG: Magneto-encephalography
PLS: Partial least squares
PoNS®: Portable neuromodulation stimulator
PT: Physiotherapy
RH: Right Hand
TBI: Traumatic brain injury
TLNS: Translingual neurostimulation
TMS: Transcranial magnetic stimulation
