# Supplementary material for: Portable neuromodulation induces neuroplasticity to re-activate motor function recovery from brain injury: a high-density MEG case study
Source: J Neuroeng Rehabil. 2020 Dec 1;17:158. doi: 10.1186/s12984-020-00772-5 (PMC7708191; doi:10.1186/s12984-020-00772-5)
Supplement: Supplementary file 1 — Additional file 1: Figure S1. Part A Five regions—randomly labelled A-E—of greatest fMRI activation change over the three-year study duration from Phase 1, showing increased activation in areas immediately posterior to damaged structures. Co-registered functional data were concatenated into a 4D volume and passed to FSL MELODIC for independent components analysis along the 4th dimension, using automatic dimensionality estimation. Resulting temporal components were averaged into years 1–3 and compared using a paired t-test. Spatial distribution maps localized the regions of greatest changing brain activity. FSL Cluster was used to extract “neuroplasticity clusters” from the lower limb IC3 map. (Refer to Part B for activation changes over time for each of these regions). All activation is thresholded for p<0.05 (corrected). Part B: Activation changes over the three-year study duration for the five clustered regions (labelled A–E) of largest overall fMRI change (shown in Additional figure Part A). Blue lines represent the activation for each voxel within the cluster, red lines show the average activation of all voxels, and the line of best fit indicated in black to show the general trend over time. [file 12984_2020_772_MOESM1_ESM.docx]

# Additional Figures


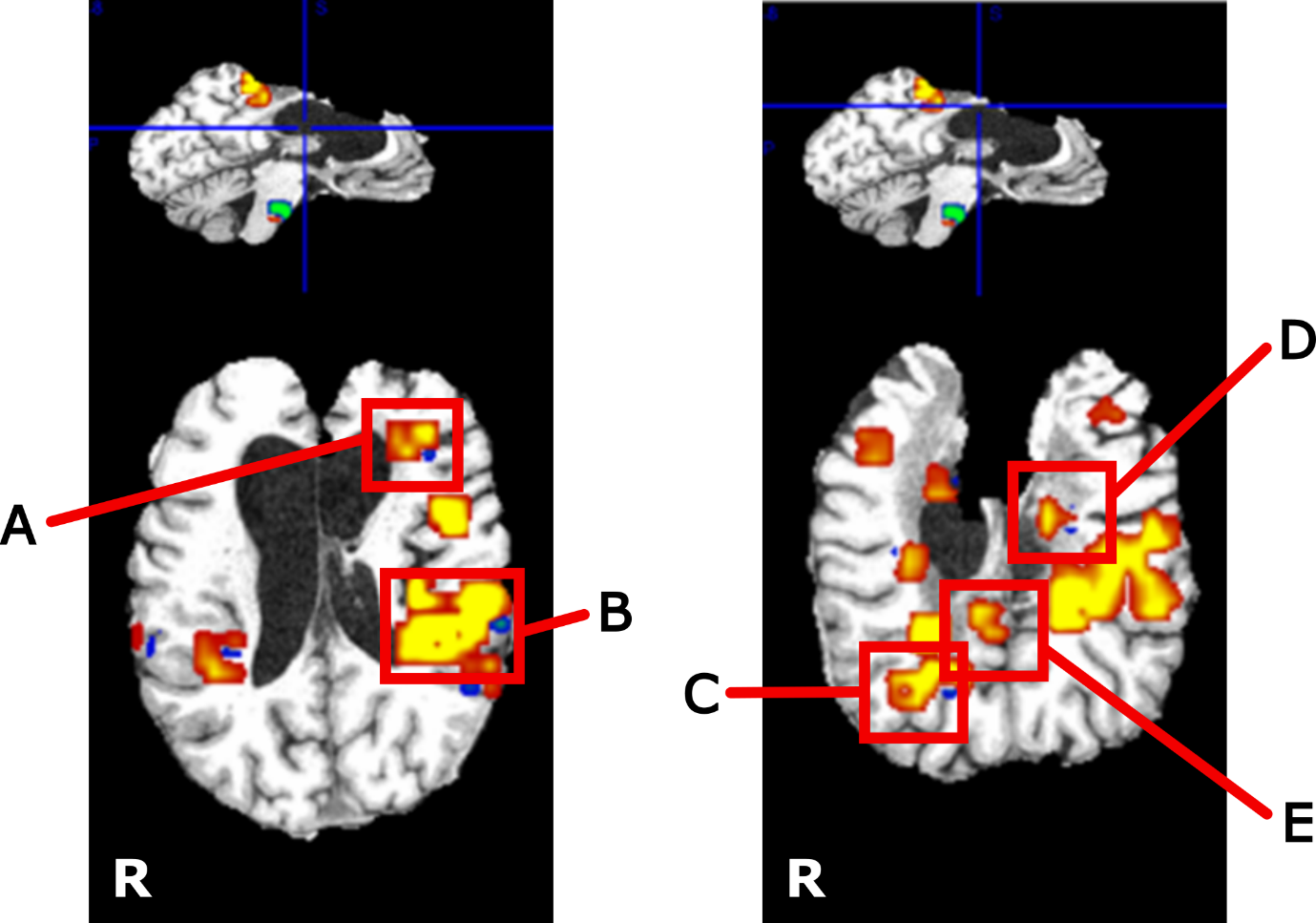


Additional Figure S1 Part A: Five regions – randomly labelled A-E - of greatest fMRI activation change over the three-year study duration from Phase 1, showing increased activation in areas immediately posterior to damaged structures. Co-registered functional data were concatenated into a 4D volume and passed to FSL MELODIC for independent components analysis along the 4^th^ dimension, using automatic dimensionality estimation. Resulting temporal components were averaged into years 1-3 and compared using a paired t-test. Spatial distribution maps localized the regions of greatest changing brain activity. FSL Cluster was used to extract “neuroplasticity clusters” from the lower limb IC3 map. (Refer to Part B for activation changes over time for each of these regions). All activation is thresholded for p<0.05 (corrected).


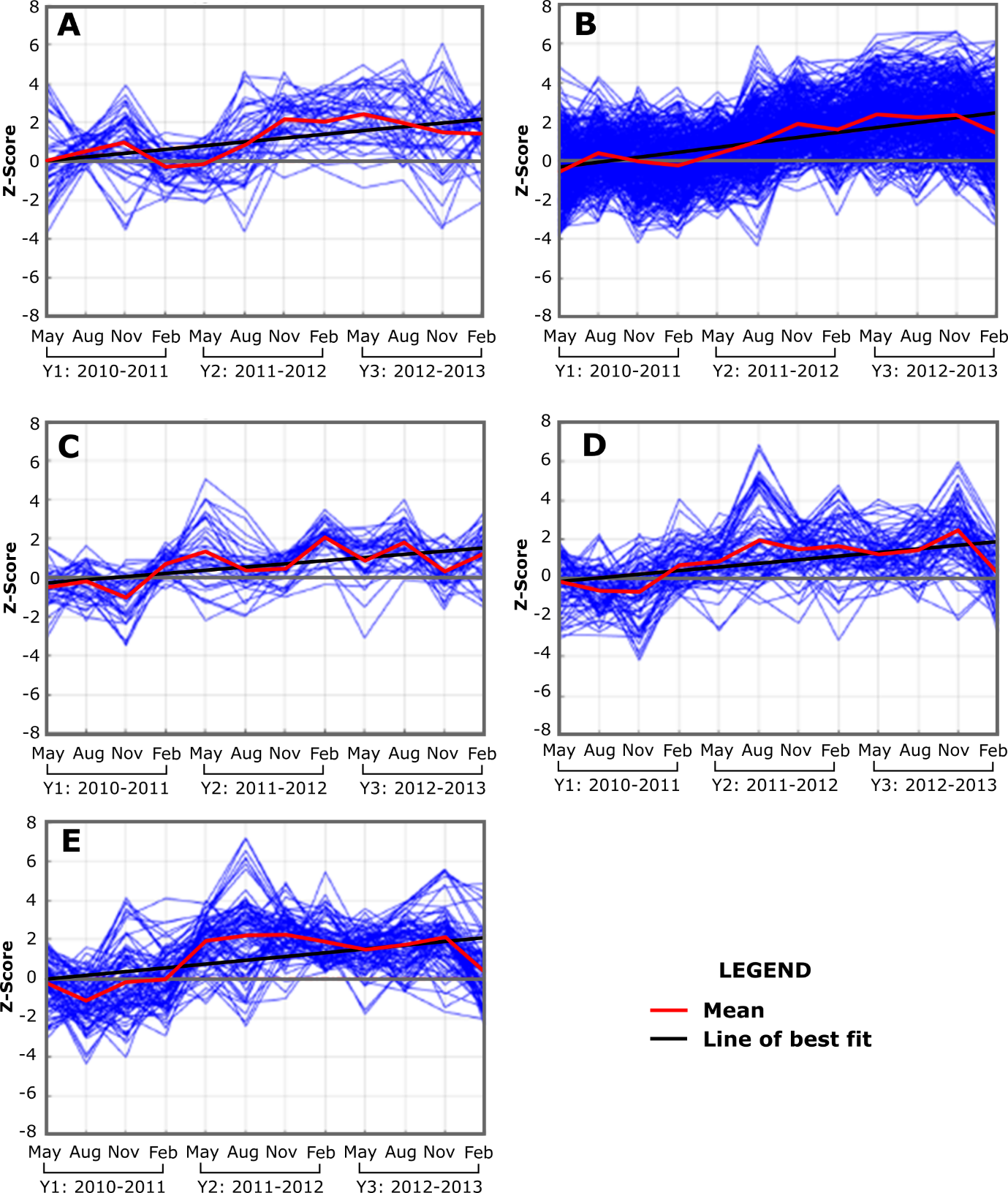


Additional Figure S1 Part B: Activation changes over the three-year study duration for the five clustered regions (labelled A-E) of largest overall fMRI change (shown in Supplemental Figure Part A). Blue lines represent the activation for each voxel within the cluster, red lines show the average activation of all voxels, and the line of best fit indicated in black to show the general trend over time.
